# The Survival Benefit of Postoperative Bacterial Infections in Patients With Glioblastoma Multiforme: Myth or Reality?

**DOI:** 10.3389/fneur.2021.615593

**Published:** 2021-02-05

**Authors:** Syed Faraz Kazim, Erick Martinez, Tyler J. Hough, Benjamin Q. Spangler, Christian A. Bowers, Muhammad Omar Chohan

**Affiliations:** ^1^Department of Neurosurgery, University of New Mexico Hospital (UNMH), Albuquerque, NM, United States; ^2^School of Medicine, New York Medical College (NYMC), Valhalla, NY, United States; ^3^School of Medicine, University of New Mexico (UNM), Albuquerque, NM, United States; ^4^Department of Neurosurgery, University of Mississippi Medical Center (UMMC), Jackson, MS, United States

**Keywords:** glioblastoma multiforme, bacterial infections, survival benefit, mechanism(s), immune response

## Abstract

Glioblastoma multiforme (GBM), the most common malignant brain tumor, universally carries a poor prognosis. Despite aggressive multimodality treatment, the median survival is ~18–20 months, depending on molecular subgroups. A long history of observations suggests antitumor effects of bacterial infections against malignant tumors. The present review summarizes and critically analyzes the clinical data providing evidence for or against the survival benefit of post-operative bacterial infections in GBM patients. Furthermore, we explore the probable underlying mechanism(s) from basic science studies on the topic. There are plausible explanations from immunobiology for the mechanism of the “favorable effect” of bacterial infections in GBM patients. However, available clinical literature does not provide a definitive association between postoperative bacterial infection and prolonged survival in GBM patients. The presently available, single-/multi-center and national database retrospective case-control studies on the topic provide conflicting results. A prospective randomized study on the subject is clearly not possible. Immunobiology literature supports development of genetically modified bacteria as part of multimodal regimen against GBM.

## Introduction

Glioblastoma multiforme (GBM), the most common malignant brain tumor, universally carries a poor prognosis (1–3). The current standard treatment for newly diagnosed GBM include maximal surgical resection, followed by radiation therapy given concomitantly with temozolomide (TMZ) followed by adjuvant TMZ chemotherapy (4–6). Despite aggressive multimodality treatment, the median survival is ~18–20 months, depending on molecular subgroups (2, 7, 8). Extent of resection, adjuvant chemo-radiation, young age, high Karnofsky Performance Score (KPS), and DNA repair enzyme *O*^6^-methylguanine-DNA methyltransferase (MGMT) promoter hypermethylation are factors associated with improved survival (9–11).

It has long been speculated that postoperative bacterial infection may prolong survival in cancer patients by stimulating immune response (12). William B. Coley, an orthopedic surgeon and pioneer of cancer immunotherapy, was the first to extensively study the role of bacterial inoculants in cancer progression during the late nineteenth and early twentieth centuries (13–15). In 1891, he injected streptococcal organisms into a patient with inoperable cancer, and was able to show significant tumor shrinkage (15). Over the next 40 years, as head of the Bone Tumor Service at Memorial Hospital in New York, Dr. Coley injected more than 1,000 cancer patients with bacteria or bacterial products, known as Coley's Toxins, and reported favorable results, especially in bone and soft-tissue sarcomas (15). Later on, during mid-twentieth century, Nauts et al. showed that several bacterial organisms and their products could induce tumor regression in malignant tumors in humans (13). In late twentieth century, with the universal adoption of aseptic surgical technique, advent of antibacterial pharmacotherapy, and the emergence of adjuvant chemo-radiation, interest in natural, iatrogenic, or induced infection as a therapeutic approach for patients with cancer fell out of favor (16, 17).

While there is a long history of observations suggesting antitumor effects of natural bacterial infections against malignant tumors (18), conflicting clinical neurosurgical evidence exists regarding the effect of post-operative bacterial infections on survival in GBM patients which precludes any definitive conclusion(s) (19–21). The present review summarizes the clinical data on the role of post-operative bacterial infections on survival in GBM patients. Furthermore, we explore the probable underlying mechanism(s) from basic science studies on the topic. The mechanistic insights from basic science provide a rationale for bacterial pathogens-related antitumor effect.

## Clinical Studies on the Potential Role of Postoperative Bacterial Infection on Survival in GBM Patients

Post-operative bacterial infections following surgical resection of GBM offer a passive conduit to study the role of pathogen-induced immunologic responses in tumor progression. However, the role of postoperative infection on survival in patients with GBM has not been rigorously studied. Our literature search identified one national database retrospective case-control study, one multi-center, retrospective case-control study, three single-center, retrospective case-control studies, and few case reports/series on the topic (Table 1). A recent multi-center, retrospective case-control study reported a significantly shorter survival in GBM patients with infections after surgery for primary tumors (median survival 381 days/12.5 months) as compared to control group (median survival 547 days/18 months) (22). Majority of infections in the study by Salle and colleagues were deep wound infections (22). Another study by Bohman et al. (19) in 2009, did not find a statistically significant survival advantage in 17 patients with postoperative infection after GBM resection (median survival, 13.1 months), out of 383 patients included in their single-center retrospective study. However, a moderate, statistically insignificant survival advantage was seen in the infected group, however, when patients with infections in the first quarter and first half of their postoperative survival period were analyzed, this survival advantage disappeared (19). There was no significant survival difference in any subgroup assessed, including deep infections, bone flap infections, or infections caused by any specific organism (19).

**Table 1:** Literature review of effect of postoperative bacterial infections on survival in GBM patients.

**References**	**Type of study**	**No. of patients with GBM**	**Major infection type**	**Major offending organism**	**Survival (months)**	**Outcome**
Salle et al. (22)	Retrospective case-control	64	Deep incisional wound infection = 25 Organ wound infection = 39	*S. aureus* = 20 *S. epidermidis* = 14 *P. acnes or C. acnes* = 5 *Corynebacterium* = 3 *E. cloacae* = 3 *S. capitis* = 3	12.5^†^	Significantly shorter survival in GBM patients with postoperative infections
Chen et al. (21)	Retrospective case-control	154	Not reported	Not reported	5^†^	No significant survival advantage
De Bonis et al. (20)	Retrospective case-control	10	Abscess = 8 Wound/bone = 5	*S. aureus* = 6 *S. epidermidis* = 2 Gram positive = 2	30^†^	Significant survival advantage
Bohman et al. (19)	Retrospective case-control	17	Wound = 8 Abscess = 6 Wound/bone = 4 Meningitis = 3	*S. epidermidis* = 6 *P. acnes or C. acnes* = 6 *S. aureus* = 4	13^†^	No significant survival advantage
Alexiou et al. (23)	Case series	2	Wound infection	*S. hemolyticus* = 1 *S. epidermidis* = 1	42, 168	Survival advantage
Bowles and Perkins (24)	Case series	4	Wound/bone infection	*S. aureus* and E. *aerogenes* = 2 *E. aerogenes* = 1	120^*^	Survival advantage
Walker and Pamphlett (25)	Case report	1	Extra/subdural fluid collection, wound infection	*S. epidermidis*	60	Survival advantage

† *Median time*.

* *The study followed up cases up to 10 years (120 months)*.

*C. acnes, Cutibacterium acnes; E. aerogenes, Enterbacter aerogenes; E. cloacae, Enterobacter cloacae; P. acnes, Propionibacterium acnes; S. aureus, Staphylococcus aureus; S. capitis, Staphylococcus capitis; S. epidermidis, Staphylococcus epidermidis; S. hemolyticus, Staphylococcus hemolyticus*.

In contrast, a single-center, retrospective study by De Bonis and colleagues reported a survival benefit of post-operative bacterial infections in GBM patients (20). This study evaluated 10 post-operative surgical site infections in 197 GBM patients (20). Kaplan-Meier analyses revealed a significant survival advantage in infected patients (median survival 30 vs. 15 months in controls, Breslow test, *p* < 0.01 and log-rank test, *p* = 0.01) (20). The cumulative survival probabilities at 1, 2, and 3 years for patients with and without postoperative infection were 100 and 42%, 67 and 22%, and 37 and 10%, respectively (20). All patients had infections during the first quarter of their overall survival period, with *Staphylococcus aureus* being the most common pathogen (6 cases, 60%) (20). Five patients had intracranial abscess; 3 patients had both abscess and surgical wound infection; and 2 patients had surgical wound and bone flap infection requiring surgical revision (20). The analysis of survival by pathogen and site of infection did not show any significant differences, owing to small sample size (20).

In a recent population based study, Chen et al. (21) analyzed 3,784 patients with GBM from the linked Surveillance, Epidemiology, and End Results (SEER)–Medicare database. They found that 154 (4.2%) patients had infection (defined as bacterial or non-bacterial meningitis, intracranial abscess, or postoperative infection by ICD-9 coding) within the first month of surgery (21). In patients with GBM who had an infection within 1 month of surgery, there was no significant difference in survival (median, 5 months) compared with patients with no infection (median, 6 months, *p* = 0.17) (21). Given limitations of the SEER database, this study could not provide further details on the type of organism or severity of infection.

In a case series, Alexiou *et al*. reported a survival time of 42 months in a GBM patient with postoperative bacterial infection, with an institutional median survival of 15.5 months (23). Another GBM patient in the same series with postoperative bacterial infection had an overall survival time of 14 years (168 months) (23). In two separate case report/series, survival times of 120 and 60 months, respectively, have been reported with postoperative bacterial infections (24, 25).

Antitumor effects of bacterial infections have also been described in central nervous system (CNS) tumors other than GBM. For example, regression of a skull base liposarcoma was reported after surgical site infection with *Corynebacterium hemolyticum* and *Staphylococcus epidermis* (26). There are also clinical studies outside the neurosurgical literature which hint toward a probable association between infection and survival in tumors. Ruckdeschel et al. (27) in a retrospective study evaluated 18 patients with lung cancer complicated by postoperative empyema. The 5-year survival rate for the empyema group was 50% as compared to 18% for the control group consisting of 34 patients (27). In another study, Jeys and colleagues evaluated the effect of postoperative infection in patients treated for osteosarcoma, using endoprosthetic replacement and neo-adjuvant chemotherapy (28). The study reported that patients with osteosarcomas who had postoperative infection had a survival rate of 84.5% at 10 years (*n* = 41) compared to 62.3% in the noninfected group (*n* = 371; *p* = 0.017) (28). It was found that infection was an independent prognostic factor in Cox regression analysis (28). Miller and Nicholsen reported on 52 cases of reticulum cell sarcoma of bone and found tumor regression with prolonged survival associated either with infection or with treatment by bacterial toxin therapy (29). Older studies similarly found association between infection and improved survival in melanoma (30) and acute lymphoblastic leukemia (31). Finally, a canine study of limb-salvage surgery for osteosarcoma similarly reported survival advantage of post-operative infection (32).

## Methodological Constraints in Available Clinical Literature

Several methodological issues likely explain conflicting results from the clinical studies described above. First, a retrospective study design does not allow for the control of many variables such as inconsistent treatment of post-operative bacterial infection. Although discussed as confounders, the near-universal use of peri-operative steroids and adjuvant chemo-radiation should ideally not be seen as confounder in the modern era. Nonetheless, it is imperative to mention here the steroid-induced systemic immunosuppression and neutrophil demargination as well as direct radiation-induced toxicity to tumor resident immune cells. These effects of peri-operative steroids and adjuvant chemo-radiation can be expected to confound the survival benefit of infection on survival in GBM patients. Also, in patients treated for recurrent GBM, i.e., those who have failed standard of care adjuvant treatment, the use of many second-line agents, including immune-modulators [such as bevacizumab, a monoclonal antibody that binds to vascular endothelial growth factor (VEGF) and inhibits the growth of tumor blood vessel] are likely to act as potential confounders. Another variable is the severity of infection, i.e., superficial vs. deep. De Bonis et al. (20) reported a significant survival benefit of deep post-operative infections. A similar trend was reported by Bohman et al. (19). One could argue that involvement of resection cavity would provide the best probability for tumor antigen presentation to host lymphocytes and the highest likelihood of a targeted antitumoral immune response (19).

A major issue with clinical studies evaluating possible survival benefit of post-operative bacterial infections is the rarity of post-operative infections (given the sterile techniques used and the widespread antibiotics prophylaxis in present day neurosurgical practice) produces a small case group, which reduces the power of any study. For instance, the study by De Bonis et al. (20) identified 197 patients with primary GBM during a 7-year retrospective study period, and only 10 out of 197 (5.08%) patients had post-operative bacterial infections. Similarly, the study by Chen and colleagues evaluating a national database of 3,784 GBM patients reported that 154 (4.2%) patients had post-operative infection (21).

The most common organisms in the studies by Salle et al. (22), De Bonis et al. (20) and Bohman et al. (19) were gram positive bacteria (*Staphylococcus aureus* and *Staphylococcus epidermidis* in the first two studies, and *Staphylococcus epidermidis* and *Propionibacterium acnes* in the third one). Salle et al. (22) reported a significantly shorter survival time in patients with postoperative bacterial infections as compared to controls, De Bonis et al. (20) reported survival benefit and Bohman et al. (19) didn't find any survival advantage with infection. Another smaller case series reported a survival benefit in GBM patients with gram positive bacterial infections (23). While the largest clinical study on topic didn't report the type of infections involved (21), another series reported survival benefit after infection with a Gram negative organism, *Enterobacter (Klebsiella) aerogenes* (24). While Coley's studies focused primarily on survival benefit of Gram positive (erysipelas) toxins, the beneficial effect of therapeutic strategies utilizing regimens based on Gram negative bacteria-associated lipopolysaccharide both in *in vitro* and *in vivo* animal model studies against glioma cells (33, 34) support the notion of survival benefit induced by Gram negative infections also.

## Mechanism(s) Implicated in Survival Benefit of Bacterial Infections in GBM

**Figure 1:**
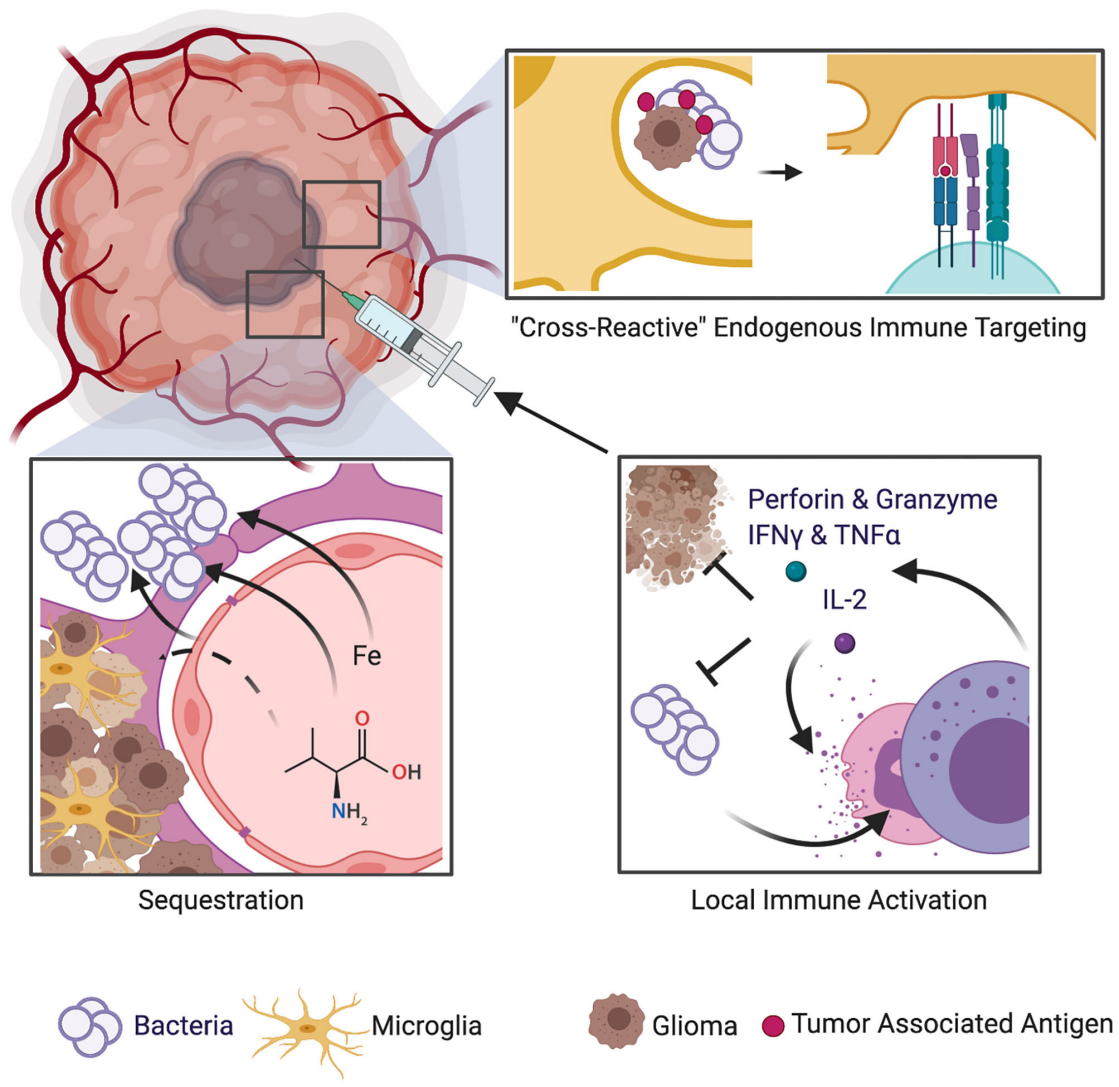
Schematic of mechanisms implicated in potential survival benefit of bacterial infections in GBM. Several theories have been proposed to explain how bacterial infection could lead to improved survival in GBM patients. One theory suggests that bacteria employ immune-evasion techniques such as sequestration of protein products and nutrients such as iron that are vital to sustain the vasculature and immense metabolic demands of neoplastic cells. Another theory is that bacterial infection within or near the tumor bed may stimulate the patient's immune response, thus resulting in an *endogenous immune targeting* against glioma cells. A biological argument that encompasses the ability of bacterial infections to induce augmented immunostimulatory response and to evoke a cascade of cytokines and chemokines, some of which also exert anticancer effects, has also been put forth.

Several theories have been proposed to explain how bacterial infection could lead to improved survival in GBM patients. De Bonis et al. (20) hypothesized that proliferation of bacteria is likely to induce local competition for resources between the tumor cells and the replicating microorganisms. Bacteria such as *Staphylococcus aureus* employ immune-evasion techniques such as sequestration of protein products and nutrients such as iron that are vital to sustain the vasculature and immense metabolic demands of neoplastic cells. Bohman et al. (19) suggested that infection within or near the tumor bed may stimulate the patient's immune response, thus resulting in an *endogenous immune targeting* against glioma cells. A biological argument that encompasses the ability of bacterial infections to induce augmented immunostimulatory response and evoke a cascade of cytokines and chemokines, some of which also exert anticancer effects, has been put forth (20). Modern biotechnology is harnessing this therapeutic potential by employing genetically modified bacteria to fight cancer (18, 35). Figure 1 provides a schematic of potential mechanisms of beneficial effect of post-operative bacterial infections on survival in GBM patients. The intrinsic CNS immune milieu [comprising of macrophages (36, 37) and glymphatic system (network of vessels that clear waste from the CNS) (38)] is subverted by growing GBM, and in this regards, the *endogenous immune targeting* against GBM cells by bacterial infections can exert a beneficial effect.

As mentioned above, local release of cytokines and activation of cancer-targeting immune cells could be the main mechanism by which bacterial infection may increase survival in GBM. The intracavitary introduction of interleukin-2 (a cytokine predominantly secreted by activated T- cells and a key player in the cell-mediated immune response) and lymphokine-activated killer cells was reported to improve survival in patients with recurrent malignant glioma (39). Interleukin-2 gene therapy was also found to be beneficial in a GBM patient (40). Similarly, tumor necrosis factor alpha (TNFα, an inflammatory cytokine produced by macrophages/monocytes during acute inflammation that is responsible for a diverse range of signaling events within cells, leading to necrosis or apoptosis), was found to reduce GBM growth both *in vitro* and *in vivo* (41, 42). Cytokine-targeted immunotherapy has been identified as a potential therapeutic strategy for gliomas and is an active area of research in the neuro-oncology field (43–45). For example, reducing expression of tumor cell-secreted cytokine inhibitors induced apoptosis of glioma cells (44). A number of bacteria associated antigens could act as immunostimulatory agents against proliferating glioma cells (23, 24).

The hypothesis of bacterial toxins-mediated immunotherapy for GBM is indirectly supported by observations in animal models and humans. For instance, a study reported that intracerebrally implanted heat-inactivated staphylococcal epitopes mixed with 9L gliosarcoma cells in Wistar rats, led to significantly prolonged survival than experimental controls (46). In a study by Tanaka and colleagues, Picibanil, also called OK-432 (an immunopotentiator and a low virulent mutant strain of Lancefield's Type III, Group A *Streptococcus pyogenes*) was injected intratumorally in 13 patients with malignant brain tumors, resulting in significant tumor regression in 50% of patients (47). Further indirect evidence comes from basic science studies of antitumor effects of bacterial toxins. An investigation by Duong and colleagues found that toxins from bacteria can directly induce apoptosis in tumor cells and increase neutrophil recruitment to the tumor microenvironment (TME) (35, 48). Bacterial toxins can also upregulate connexin 43 (a ubiquitous protein that forms gap junctions and that is normally lost during tumor progression), which in turns creates bacterial-induced gap junctions between tumor cells and dendritic cells and allows for cross-presentation of tumor antigens to the dendritic cells (49). This ultimately activates cytotoxic T cells against the tumor to limit its growth (49). Similarly, bacterial flagellin can also increase the antitumor response of CD8^+^-cells (50).

The immunologic evasion of GBM is well documented; several blocking factors are produced by this type of brain tumor which suppress cytotoxic lymphocytes (3, 51). Higher expression of Immune Checkpoint (IC) molecules such as PD-L1 which provide inhibitory signals to T cells has been documented (52, 53), and IC Inhibitors (ICI) are presently being evaluated as potential GBM treatment in clinical trials (54). These data support the concept of immune evasion by GBM. It seems likely from preclinical and clinical data bacterial infections elicit some immunological cross-reactive attack that is directed not only against the infectious agent but also against the tumor cells. This augmented, “cross-reactive” immunostimulatory response to subvert infection, also has antitumor effect.

## Conclusion

Historical evidence suggests beneficial effect of bacterial infections in tumor regression in general. There are plausible explanations from immunobiology for the mechanism of this potential anti-tumor activity in GBM patients. However, the available clinical neurosurgical literature fails to confirm such an association and a prospective randomized study on the topic is clearly not possible. While available clinical studies do not provide definitive association between post-operative bacterial infection and prolonged survival, immunobiology literature strongly suggests exploring genetically modified pathogens in a multimodality treatment approach for GBM.

## Author Contributions

SK, EM, TH, BS, CB, and MC, wrote the initial draft and edited the final version of the manuscript. MC and CB provided overall supervision for the manuscript. All authors contributed to the article and approved the submitted version.

## Conflict of Interest

The authors declare that the research was conducted in the absence of any commercial or financial relationships that could be construed as a potential conflict of interest.
